# To the lighthouse: navigating nephrology through the world of social media

**DOI:** 10.1093/ckj/sfae170

**Published:** 2024-06-20

**Authors:** Lauren Floyd, Jasmine Sethi, Madelena Stauss, Alexander Woywodt

**Affiliations:** Department of Renal Medicine, Lancashire Teaching Hospitals NHS Foundation Trust, Preston, Lancashire, UK; Post Graduate Institute of Medical Education and Research, Chandigarh, India; Department of Renal Medicine, Lancashire Teaching Hospitals NHS Foundation Trust, Preston, Lancashire, UK; Department of Renal Medicine, Lancashire Teaching Hospitals NHS Foundation Trust, Preston, Lancashire, UK

## INTRODUCTION–#NEPHROLOGY

Back in 2009, two articles in *NDT Plus*, the predecessor of *CKJ*, described opportunities for using the internet [1, 2]. A decade and a half later, this practice has gone viral. We live and work in an ever-expanding ‘socialverse’, with >5 billion people globally using social media (SoMe) platforms, partly as professional and educational tools. However, adoption remains low in some low- and middle-income countries (LMICs) [3], with some studies suggesting that higher tweet numbers can be a proxy for gross domestic product (GDP) [4]. Risks also exist and regulations range from non-existent to minutely prescriptive, with serious sanctions if guidance is breached [5]. So, in an age of instant information, with a multiverse of options available at a single touch, how best to navigate through this world of opportunity and risk? Here, we provide a brief primer on SoMe in nephrology, focusing on advantages, pitfalls and tips for maximizing utility for clinicians. Finally, we highlight a list of challenges for the next decade, which will undoubtedly take us on a journey to an entirely new realm of connectivity.

## X-CITING PROSPECTS—WHAT ARE THE BENEFITS OF SoMe?

From vlogs, posts and podcasts to blogs, livestreams and content platforms, there is huge variety not only in terms of content and format, but also with regards to the target audience. The widespread availability of SoMe means that information can be disseminated globally, quickly and freely. This was particularly evident in the COVID-19 pandemic, with an unprecedented trend towards sharing and disseminating research immediately and globally via SoMe [6]. This development has paved the way for a changed research landscape where, in theory, medical information is universally accessible not only for healthcare providers, but also for patients, laypeople, students and those without economic means.

SoMe has incredible power to engage and connect users and thereby foster intra- or cross-speciality networking and collaboration among researchers, patient partnerships, charities, invested stakeholders and global organizations. For example, in solid organ transplantation where SoMe has offered broad outreach potential, a meta-analysis from 2009 showed there was a 5% increase in registry sign-ups across 23 studies that used media campaigns. However, this is not without concerns regarding coercion, organ trading and significant ethical implications [7].

SoMe also helps with recruitment, and institutions such as Yale and Johns Hopkins University have used SoMe to promote fellowship applications, jobs and research opportunities. Overall, SoMe has been shown to accelerate recruitment and enhance diversity in the workforce, as well as facilitate remote mentorship and role modelling, which would be much less feasible without virtual connections.

The educational potential of SoMe in nephrology is equally impressive. Free open-access medical education (FOAMed) has seen exponential growth in recent years [8]. For example, the international Glomerular Disease Study & Trial Consortium (GlomCon) has >8700 YouTube subscribers and an impressive 519 600 YouTube channel views across various nephrology-related topics. A recent study described how almost three-fourths of responding American Society of Nephrology fellows utilized Twitter (now X), blogs and podcasts for up-to-date information [9].

Since 2009, >19 new nephrology-related podcasts have become available, with the most recent, ‘A pinch of salt’, from the European Renal Association (ERA). Their podcasts relate to several aspects of nephrology, such as career advice, general nephrology and nephropathology, and demonstrate the way SoMe users can customize their individual learning and development needs.

Accessing bite-sized content to facilitate ‘microlearning’ offers a broader range of relevant information compared with bulkier resources such as books and journals. Multiple sources of information can be aggregated into one SoMe thread or post so users can curate information on a topic from multiple perspectives. Educational SoMe can also be more timely and portable, and may appeal to different learning styles when compared with educational events such as congresses and workshops.

The very recent ERA Congress in Stockholm also demonstrated the power of SoMe, with nearly 9000 tweets and posts with the hashtag #ERA24 and >380 virtual views of one of the late-breaking clinical trials symposia. It highlights the flexibility and far-reaching impact that SoMe has for learners who may otherwise not be able to attend due to geography or funding. The environmental impact of avoiding face-to-face interaction and artefacts is also noteworthy [10]. However, virtual attendance and SoMe delivery of such events may limit interactions between speakers and attenders, preclude networking and create challenges in keeping participants engaged. Technical glitches and cybersecurity threats are other downsides of virtual attendance at conferences.

The ability of SoMe to stimulate instant discussion is unrivalled by any of the traditional resources. What would previously have relied on letters to the editor now occurs contemporaneously and with a diverse, enthusiastic and knowledgeable audience. The timing of engagement is also more flexible, and users can choose between asynchronous platforms, such as blogs or podcasts, and synchronous active participation, such as livestreaming or posting from a conference. In addition, journals now offer altimetrics on published articles, which creates an attention score derived from the impact that publications have on SoMe and news outlets. For example, one of *CKJ*s most successful published articles was ‘Who killed Bruce Lee? The hyponatremia hypothesis’. While this article has had few citations, it has been covered by >190 news outlets and has been mentioned in nearly 400 tweets [11]. Overall, this publication was therefore in the top 5% of research outputs scored by Altimetric [11]. The value of these post-publication metrics allows nephrology journals to track what is engaging users and stimulating debate, with further published research focusing on controversial topics or those requiring clarification. The impact of these newer methods is not yet known, with the ‘number needed to tweet’ for meaningful effect still up for debate.

## GET THE ’GRAM—HOW CAN A NEPHROLOGIST GET THE MOST OUT OF SoMe?

Keeping professional and personal accounts separate is a good way of avoiding some of the common online pitfalls. Establishing a professional profile allows you to share research and professional opinions freely and for colleagues to follow your updates. By liking and following professionals or organizations, your algorithms become tailored to the renal specialty, enhancing the relevance of your content and increasing your visibility, making it easier for others to discover and connect with you as well. However, it is important to remember that your intended audience cannot be guaranteed and that all posts can be seen by patients and their families.

By diversifying SoMe platforms, you can access varied and unique content depending on specific learning needs and preferences. YouTube has great lectures and revision resources, while X can keep you up to date with recently published papers and clinical trials. Using the relevant save, like and bookmark options is helpful in cataloguing interesting content to come back to for further reading or referencing and can save a lot of unnecessary scrolling time.

E-seminars, podcasts and webinars are typically free and often represent excellent educational resources with the added opportunity to contact expert clinicians and ask questions. Following relevant hashtags, such as #CKD or #Dialysis can help access a broad range of content and increase the visibility of relevant information. Engagement by contributing to online discussions, posts or comments can be a great way to learn and participate in critical thinking. Interactive activities such as online polls or quizzes can promote active engagement and participation and make the learning process more enjoyable. #NephMadness is an educational initiative that uses gamification to facilitate discussion and learning around hot topics in nephrology while using a tournament format to facilitate competition. Look for other SoMe platforms that reward users with badges, points or leadership boards to make learning more entertaining.

**Figure 1:**
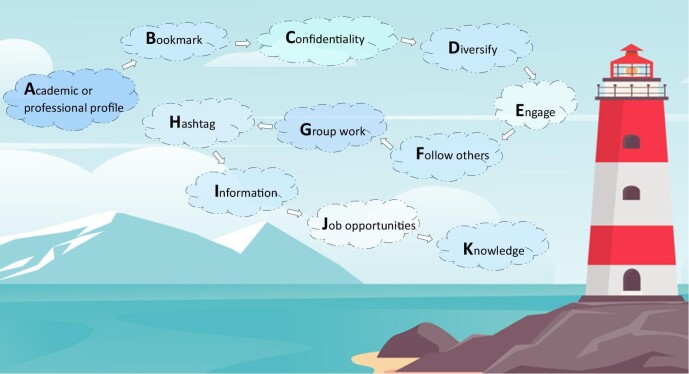
All in the cloud: the A to K of making the most of social media. Background obtained commercially from Vectorstock Ltd., April 2024, by A.W.

Discussion and networking through SoMe with peers, experts and organizations is an excellent method for developing collaborative relationships and knowledge sharing. Using your name in posts aids transparency and we would recommend declaring any conflicts of interest where relevant as well. It is crucial to prioritize privacy, respect professional boundaries and uphold patient confidentiality. As a guide, one should apply the same standards online that one might use in face-to-face interactions. This includes respectful engagement, even amidst differing viewpoints [12]. Suggestions on how to use SoMe for professional development and learning are summarized in Fig. 1.

## FEELING TWITCH-Y? POTENTIAL RISKS AND CHALLENGES WITH SoMe USE IN NEPHROLOGY

One concern about SoMe content is the lack of quality control. Without the formality of traditional peer review, there is a risk that the information may be based on anecdotal evidence or opinion rather than robust data. Going forward, adding some form of quality control will be key for educational SoMe material, including content generated by artificial intelligence (AI). In addition, the integration of virtual reality into SoMe platforms in the coming decade requires careful consideration of opportunities and challenges, including that of virtual impostors.

Different SoMe platforms can provide variety, but the scattering of information can make it difficult to obtain a comprehensive understanding of a topic. Some form of registry or link between related content would be very useful, where content can be collated from different SoMe sources. Algorithms can be beneficial for suggesting new areas of discovery based on past preferences, but overly targeted suggestions can miss important information and create echo chambers, leading to bias and skewed perspectives. The lack of diversity in traditional publications is already documented, and similar concerns may apply to SoMe.

Formal author recognition for content on SoMe is also lacking at present, even though some content reaches a bigger audience than a comparable article in medical journals. It is likely that in the near future some form of SoMe altimetric will be formally recognized to measure output. With the formalization of SoMe content, bias and conflict of interest are also concerns. Authors should perhaps be required to publish conflict-of-interest declarations akin to those used by journals and other online content. Similar concerns apply to commercial interests, with some believing contemporary SoMe platforms are less about community and more about connecting brands with customers [13].

While most users are aware of issues around patient consent and confidentiality and know to avoid posting patient identifiable content or to seek formal consent, more subtle risks exist. For example, describing anonymized case vignettes may still identify the patient in which the condition is very rare or the presentation is very unusual. Such unintentional breaches of confidentiality can erode trust in healthcare institutions and lead to disciplinary action and legal consequences. This aspect of SoMe is increasingly regulated by national institutions and employers and is often now taught in undergraduate medical studies. SoMe guidelines are still far from universal and often focus on the risks of SoMe use. Most guidance documents encourage the use of SoMe overall [12], but there is often very little practical guidance. On the other hand, some guidelines are very detailed and prescriptive [5].

Copyright issues can also be raised, and while referencing guidelines provide recommendations on how to formally cite tweets and other SoMe content, it should be noted that some permission requests may be required. Moreover, issues can occur such as when individuals share copyrighted content material without attribution. Users should use/share open-access resources whenever possible, respect formal guidelines, obtain permission from copyright holders and provide proper attribution.

Professional boundaries are also important during interactions with both peers and patients. Many physicians value patient engagement, but most institutions now advise caution. Potential issues typically include patients seeking medical advice or voicing criticism via SoMe. Although less common, harassment and fraud do also occur and ensuring safe and enjoyable interactions is a key challenge going forward. Persistent online criticism can tarnish a physician’s reputation, erode patient trust and lead to post-traumatic stress disorder (PTSD). It is key to remain courteous and avoid making insensitive remarks, and most SoMe platforms provide tools to manage interactions or raise concerns.

Mental health is not only affected by interuser interactions, but work–life balance and burnout are also relevant concerns. Endless scrolling can feel overwhelming, affect sleep and relationships and risks ‘bringing work home’ by blurring personal and professional time. Fear of missing out can also be generated by SoMe, or propagation of feelings of imposter syndrome. It is therefore essential to remain conscious of the time spent in the ‘socialverse’.

Finally, we should consider barriers to SoMe use in LMICs, although it is equally important to emphasize that this is not a universal problem. According to recent World Bank data, internet use in LMICs ranges from 6% of the population in Burundi to 91% in Kazakhstan [14]. It is difficult to formulate a workable solution for increasing access to SoMe in low-uptake countries, but initiatives by national renal societies and larger hospitals may help provide access to larger numbers of nephrologists in these countries.

Table 1 highlights the key challenges around SoMe that the renal community should address soon.

**Table 1:** SoMe in nephrology: 10 challenges for the renal community.

Challenge	How can we address the issue?
Quality control of educational content	Work within the specialty and with others on mechanisms for quality control. Standardized formats (e.g. tweetorial, visual abstract) with minimum requirements. Referencing and copyright acknowledgement need to be considered and things such as Quick Response (QR) codes may facilitate the rapid accessibility and tracking of research data and publications.
Educational activities on SoMe not formally recognized for career development	Recognition and indexing in databases such as ResearchGate, ORCID, PubMed
Bias and conflict of interest (COI)	Publish COI declarations; aim for a repository of COI for regular providers of content on SoMe. COI statements can also be included in the bio of SoMe profiles for full transparency.
Commercial interest	Vigilance and transparency incorporation in SoMe guidelines
Lack of accepted SoMe governance	Cooperation with institutions (e.g. the International Committee of Medical Journal Editors)
Content generated by AI	Content generated by AI should be identified as such; guidelines should cover this issue
SoMe will likely include virtual reality within the next decade	Conscious reflection on risks and challenges with this technology
Content is often scattered across platforms	Coordinate SoMe platforms and link similar content. Apps to consolidate content from across different platforms in one place can help to coordinate learning.
Should we interact with our patients via SoMe and, if so, how can the interaction be made more productive?	Dialogue with patients and patient interest groups and formal guidance
The renal community should aim to actively shape the future of its SoMe	Provide room for discussion at congresses and other meetings both in person and in virtual format. Develop SoMe committees and leadership.

## CONCLUSION—THE END OF THE THREAD?

**Figure 2:**
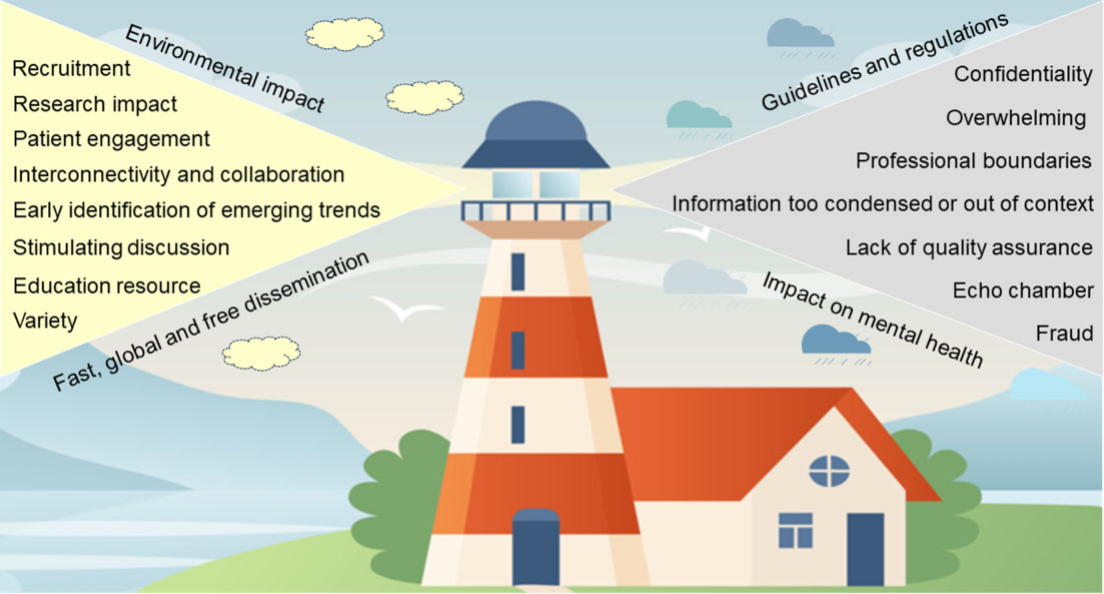
Opportunities (left) and pitfalls (right) when navigating the SoMe landscape. Background obtained from Vectorstock Ltd. under standard license (purchased April 2024). Background obtained commercially from Vectorstock Ltd., April 2024, by A.W.

Virginia Woolf's 1927 novel ‘To the Lighthouse’ [15] describes the complexities during a period of profound and lasting change and with strong societal currents. A century later, the SoMe landscape in 2024 is evolving just as rapidly and our specialty is part of this ongoing change. Navigating these opportunities and challenges will be a key skill for future generations of nephrologists. In 2024, rapid dissemination of research, recruiting an enthusiastic co-author or finding help with a complex patient online is just as easy as jeopardizing a career through a misrepresented statement. Fig. 2 summarizes the advantages and disadvantages of navigating the SoMe world. Among the many symbols in Woolf's novel, the lighthouse relates to good and bad happening to its characters—mirroring both the opportunities and risks with the use of SoMe.

## References

[1] Ande P, Chiu D, Rayner S et al. What's on the web for nephrology? NDT Plus 2009;2:119–26. 25949306 10.1093/ndtplus/sfn215 PMC4421367

[2] Chiu D, Ande P, Coward RA et al. The times they are a changin’—the internet and how it affects daily practice in nephrology. NDT Plus 2009;2:273–7. 25984013 10.1093/ndtplus/sfp041 PMC4421240

[3] Sharma S, Tannor EK, Duarsa R et al. Twitter as educational tool: a global web-based cross-sectional study on social media behavior of nephrologists. Kidney Int Rep 2023;8:2469–73. 10.1016/j.ekir.2023.08.009 38025224 PMC10658263

[4] Indaco A . From twitter to GDP: estimating economic activity from social media. Reg Sci Urban Econ 2020;85:103591. 10.1016/j.regsciurbeco.2020.103591

[5] General Medical Council . Good medical practice. https://www.gmc-uk.org/professional-standards/professional-standards-for-doctors/good-medical-practice [accessed 2 February 2024].

[6] González-Padilla DA, Tortolero-Blanco L. Social media influence in the COVID-19 pandemic. Int Braz J Urol 2020;46:120–4. 10.1590/s1677-5538.ibju.2020.s121 32550706 PMC7719982

[7] Meena P, Kute VB, Bhargava V et al. Social media and organ donation: pros and cons. Indian J Nephrol 2023;33:4–11. 37197042 10.4103/ijn.ijn_158_22 PMC10185012

[8] Renal Fellow Network . FOAMed. https://www.renalfellow.org/foamed/ [accessed 14 April 2024].

[9] Larsen DM, Boscardin CK, Sparks MA. Engagement in free open access medical education by US nephrology fellows. Clin J Am Soc Nephrol 2023;18:573–80. 10.2215/CJN.0000000000000123 36800537 PMC10278785

[10] Stoneman S, Balmer F, Moore L et al. Meet and greet but avoid the heat: a reflection on the carbon footprint of congresses prompted by ERA2023. Clin Kidney J 2024;17:sfae062. 10.1093/ckj/sfae062 38699480 PMC11063956

[11] Oxford University Press . Who killed Bruce Lee? The hyponatraemia hypothesis. Overview of attention for article published in Clinical Kidney Journal, March 2022. https://oxfordjournals.altmetric.com/details/124478656 [accessed 6 April 2024]. 10.1093/ckj/sfac071 PMC9664576 36381374

[12] Hennessy CM, Smith CF, Greener S et al. Social media guidelines: a review for health professionals and faculty members. Clin Teach 2019;16:442–7. 10.1111/tct.13033 31144449

[13] Chen B . The future of social media is a lot less social. https://www.nytimes.com/2023/04/19/technology/personaltech/tiktok-twitter-facebook-social.html [accessed 7 May 2024].

[14] World Bank . Individuals using the Internet (% of population) – low & middle income. https://data.worldbank.org/indicator/IT.NET.USER.ZS?locations=XO [accessed 6 April 2024].

[15] Woolf V . To the Lighthouse. London: Hogarth Press, 1927.

